# Inflammation and polyendocrine metabolic ovarian syndrome: utilizing proteomics to unravel this link in adolescents

**DOI:** 10.1210/jendso/bvag203

**Published:** 2026-09-03

**Authors:** Harriet M Gunn, Jenny Hällqvist, Ivan Doykov, Wendy Heywood, Russell Viner, Katharine Steinbeck, Kevin Mills

**Affiliations:** Genetics and Genomic Medicine Department, University College London Great Ormond Street Institute of Child Health, London WC1N 1EH, UK; Academic Department of Adolescent Medicine, The Children's Hospital at Westmead, Sydney, NSW 2145, Australia; Children and Young People's Endocrinology Service, University College London Hospital, London NW1 2BU, UK; Genetics and Genomic Medicine Department, University College London Great Ormond Street Institute of Child Health, London WC1N 1EH, UK; Genetics and Genomic Medicine Department, University College London Great Ormond Street Institute of Child Health, London WC1N 1EH, UK; Genetics and Genomic Medicine Department, University College London Great Ormond Street Institute of Child Health, London WC1N 1EH, UK; Population, Policy and Practice Department, UCL Great Ormond Street Institute of Child Health, London WC1N 1EH, UK; Academic Department of Adolescent Medicine, The Children's Hospital at Westmead, Sydney, NSW 2145, Australia; Discipline of Child and Adolescent Health, The University of Sydney, Sydney 2006, Australia; Genetics and Genomic Medicine Department, University College London Great Ormond Street Institute of Child Health, London WC1N 1EH, UK

**Keywords:** polycystic ovary syndrome, polyendocrine metabolic ovarian syndrome, PMOS, inflammation, adolescents, proteomic analysis

## Abstract

**Purpose and Methods:**

Polyendocrine metabolic ovarian syndrome (PMOS) affects 10% to 26% of adolescent females. Yet, its pathogenesis is poorly understood. Proteomics is a valuable tool to explore biological pathways underpinning PMOS and identify novel diagnostic/monitoring biomarkers. We undertook deep-phenotyping discovery proteomic profiling (nanoflow 2-dimensional liquid chromatography–quadrupole time-of-flight mass spectrometry with alternating low- and elevated-energy acquisition) on urine samples from a subset of a longitudinal adolescent PMOS cohort (n = 40). We compared the urinary proteome of adolescents (12-19 years, ≥1-year postmenarche) recruited from adolescent endocrinology/gynecology clinics with PMOS or insulin resistance (IR), and community-recruited age-, sex-, and puberty-matched controls. PMOS was defined by adolescent-specific criteria (irregular menstrual cycles and hyperandrogenism). Exclusion criteria included the combined oral contraceptive pill. Results were validated in the whole cohort with a multiplexed targeted proteomic panel.

**Results:**

Discovery proteomic analysis identified 3793 proteins, of which 66 were differentially expressed in the PMOS cohort vs IR and controls (candidate PMOS biomarkers). Bioinformatic analysis revealed that almost half of the PMOS-associated biological pathways were related to inflammatory/immunological responses. The inflammatory response was the most significant biological process (*P* < .001). Targeted proteomic analysis of 89 known inflammatory-associated proteins validated this association. Multivariate analysis demonstrated a distinct inflammatory proteome in adolescent PMOS.

Twenty inflammatory-associated proteins were differentially expressed in the PMOS cohort vs IR and control groups, with notable roles in oxidative stress and neuroinflammation. Six proteins were significantly different in PMOS compared with both IR and control cohorts.

**Conclusion:**

These analyses provide insight into the pathophysiology of PMOS, highlighting inflammatory processes as a prominent feature of adolescent PMOS and identifying several candidate novel noninvasive biomarkers and drug targets that need further validation.

Polyendocrine metabolic ovarian syndrome (PMOS), previously named polycystic ovary syndrome (PCOS), is the most common female endocrine disorder and is associated with significant comorbidity and economic burden (1, 2). Increasingly, it is diagnosed and treated in adolescents, where prevalence estimates vary (3%-26%) (3). It has a complex and poorly understood etiology and pathophysiology, in part, explaining the lack of targeted interventions. PMOS was classically considered a reproductive disorder mediated by androgen dysregulation (4). However, metabolic dysfunction is increasingly recognized as fundamental. This, coupled with genetic and epigenetic influences, is likely to account for its heterogeneity, adding diagnostic challenges. Differentiating between insulin resistance (IR) and PMOS, in particular, can be complex (1, 4-7). Diagnosis is further complicated as the PMOS phenotype evolves over time during gynecological maturation. Adolescence is a key window to improve health across the lifespan. Proteomics provides an exciting, valuable, “eyes open” approach to deepen knowledge of biological mechanisms underpinning PMOS and identify novel diagnostic, monitoring, and drug-target biomarkers. No previous studies have evaluated the adolescent PMOS proteome using a noninvasive biological matrix such as urine.

## Objectives

This exploratory study aimed to

- Determine the suitability of urine for noninvasive protein identification, biomarker discovery, and biological pathway analysis;
- Undertake deep-phenotyping discovery proteomic profiling to screen the urine proteome in a pilot cohort of adolescents with PMOS in comparison to healthy control and IR cohorts to identify pathways and potential biomarkers in PMOS;
- Validate findings using a multiplexed, targeted proteomic approach in a larger cohort of adolescents with PMOS or IR, as well as a control group.

## Methods

### Participants and samples

Morning midstream-urine samples were obtained from 40 prospectively recruited adolescent females (12-19 years, ≥1-year postmenarche, normal renal function). Label-free quantitative proteomic analysis was undertaken on a subset (n = 15) to compare the proteome of adolescents with PMOS (n = 6), adolescents with IR but no evidence of PMOS (n = 3), and age-, sex-, and puberty-matched healthy controls (n = 6). Targeted proteomics were subsequently performed on the whole study population: PMOS (n = 19), IR (n = 15), and controls (n = 6).

Participants with PMOS or IR had been referred to tertiary adolescent endocrinology or gynecology clinics in the Department of Adolescent Medicine, The Children's Hospital at Westmead, Sydney, Australia (2016-2018) with suspected PMOS. All eligible participants were consecutively approached to consider participation in the study and subsequently classified into PMOS or IR cohorts following clinical evaluation. PMOS was defined by adolescent-specific criteria according to Teede/Peña's 2018-2025 published guidelines following study enrollment and clinical evaluation, by 2 study investigators independently: (1) evidence of oligo/anovulation defined as either infrequent abnormal uterine bleeding/irregular menstrual cycles for gynecological age (<21 days or >45 days if 1-3 years postmenarche), less than 8 spontaneous menstrual cycles per year or amenorrhea for more than 3 months, and (2) hyperandrogenism defined as either hirsutism, persistent and severe acne, or biochemical hyperandrogenemia based on an elevated free androgen index ≥ 6.1% (5-10). Data regarding ovarian morphology on ultrasound were captured but not considered as diagnostic criteria given their unreliability during adolescence (5-7). Other diagnoses were excluded, including hyperprolactinemia, thyroid dysfunction, late-onset congenital adrenal hyperplasia, cortisol excess, or an androgen-secreting tumour. Participants were assessed for clinical evidence of IR (ie, acanthosis nigricans) using the modified Burke scale (11). Biochemical IR was defined by fasting insulin >120 pmol/L (20 μIU/mL), 2-hour insulin >450 pmol/L (75 μIU/mL) during oral glucose tolerance testing (OGTT), Homeostatic Model Assessment for Insulin Resistance ≥3.42 based on adolescent-based population reference ranges, and whole-body insulin sensitivity index (Matsuda index) < 4.3 (12, 13). The whole-body insulin sensitivity index was calculated from OGTT glucose and insulin values as previously described (14). Control samples were obtained from Steinbeck's ARCHER study, from community settings in Australia (15). This approach provided an appropriate comparator group while avoiding recruitment of controls from a tertiary referral setting, which may have introduced additional bias. Menstrual data included menarcheal age, last menstrual period, and exclusion of biochemical hyperandrogenism. Written informed assent/consent was obtained from participants and parents/guardians. Exclusion criteria included pregnancy, combined oral contraceptive pill treatment, significant cognitive impairment, or clinical instability. Full details of clinical data collection and laboratory analysis have been published (Table 1) (16). Samples were stored at −80 °C. Proteomic analysis was undertaken at NIHR BRC UCL Great Ormond Street Institute of Child Health, UK.

**Table 1:** Clinical and biochemical parameters of adolescent participants in PMOS, control and IR cohorts

						*P* value		
Parameter	n	Whole group (n = 40)	PMOS (n = 19)	IR (n = 15)	Control (n = 6)	All cohorts	PMOS vs IR	PMOS vs control
Age and menarche								
Age at assessment, years	40	15.1 ± 1.3	15.5 ± 1.3	14.6 ± 1.5	14.8 ± 0.6	.10	.53	.69
Age at menarche, years	39	11.0 ± 1.5	10.9 ± 1.4	10.8 ± 1.2	11.5 ± 0.8	.65	.88	.37
Postmenarcheal age, years	39	3.7 ± 2.0	4.5 ± 1.8	3.7 ± 1.6	2.3 ± 0.8	.21	.20	.34
Anthropometry								
BMI *z* score	38	2.5 (1.2-3.2)	2.6 (1.7-3.5)	2.7 (2.2-3.1)	0.3 (−0.1-0.5)	<.01	.86	<.01
Waist-height ratio	35	0.59 ± 0.14	0.62 ± 0.10	0.64 ± 0.70	0.41 ± 0.03	<.001	.55	<.001
Body fat, %	33	37.5 ± 9.5	39.5 ± 9.9	41.4 ± 5.7	25.7 ± 3.1	<.001	.62	<.001
Tanner stage	38	4.53 ± 0.56	4.63 ± 0.50	4.46 ± 0.52	4.33 ± 0.82	.47	.36	.22
Blood pressure								
Systolic, mm Hg	37	121 ± 10	123 ± 10	123 ± 5	114 ± 14	.11	.87	.03
Diastolic, mm Hg	37	69 ± 10	68 ± 12	69 ± 8	70 ± 6	.86	.62	.31
HbA1c, mmol/mol	32	34.0 (32.0-36.0)	34.0 (31.5-36.0)	34.5 (31.8-37.0)	—	.50	.50	—
Insulin resistance, HOMA-IR	33	7.0 ± 3.0	6.9 ± 2.7	7.1 ± 3.5	—	.90	.90	—
Androgens								
DHT, nmol/L	29	0.33 ± 0.16	0.30 ± 0.12	0.27 ± 0.12	0.44 ± 0.25	.14	.55	
DHEAS, μmol/L	33	5.6 ± 2.6	6.4 ± 2.9	4.5 ± 1.8	—	.04	.04	—
Androstenedione, nmol/L	33	6.1 ± 3.1	6.8 ± 3.1	5.2 ± 2.9	—	.13	.13	—
17OHP, nmol/L	33	2.5 ± 1.9	2.9 ± 2.2	2.0 ± 1.6	—	.24	.24	—
SHBG, nmol/L, (NR 20-114 nmol/L)	33	15.4 (13.0-33.3)	14.7 (10.4-31.3)	18.4 (13.2-38.9)	—	.41	.41	—
Testosterone, nmol/L, NR 0.5-1.3 nmol/L	39	1.45 ± 0.85	1.86 ± 0.96	1.16 ± 0.56	0.89 ± 0.36	.01	.02	.01
Free androgen index, %, NR < 6.1%	33	9.3 ± 5.5	11.4 ± 5.5	6.6 ± 4.2	—	.01	.01	—
Puberty hormones								
Oestradiol, pmol/L	38	176 (118-258)	176 (116-247)	152 (79-300)	204 (164-244)	.70	.68	.06
LH, IU/L	38	8.4 (3.7-13.9)	13.8 (9.4-19.8)	4.8 (2.1-6.9)	6.1 (2.3-8.7)	<.001	<.001	<.01
AMH, pmol/L	32	43 ± 30	56 ± 33	27 ± 16	—	<.01	<.01	—
Inflammatory markers								
Mean	31	18.6 ± 15.1	17.7 ± 15.0	19.5 ± 15.5	—	.72	.72	—
ESR, mm/hour, Median	31	19.1 (7.0-30.0)	11.0 (7.5-31.0)	19.5 (6.5-26.3)	—	.81	.81	—
Mode	31	10.0	10.0	23.0	—	.258	.258	—
Mean	31	6.0 ± 5.9	5.2 ± 6.3	6.9 ± 5.5	—	.45	.45	—
CRP, mg/L, median	31	3.0 (3.0-5.0)	3.0 (3.0-3.0)	3.8 (3.0-11.6)	—	.14	.14	—
Mode	31	3.0	3.0	3.0	—	.55	.55	—
White cell count, 10^9^/L	31	7.2 ± 2.0	6.6 ± 2.1	7.7 ± 1.7	—	.08	.08	—
Pelvic ultrasound,*^a^* ovarian volume, mL	32	8.2 ± 3.3	8.1 ± 3.3	8.4 ± 3.5	—	.80	.80	—

Data reported as mean (SD) or median (IQR). *P* values calculated from ANOVA, Kruskal-Wallis, independent *t* tests, or Mann-Whitney U tests.

Abbreviations: AMH, anti-Müllerian hormone; BMI, body mass index; CRP, C-reactive protein; DHEAS, dehydroepiandrosterone sulphate; DHT, dihydrotestosterone; ESR, erythrocyte sedimentation rate; FSH, follicle-stimulating hormone; HbA1c, hemoglobin A1C; HOMA-IR, homeostasis model assessment for IR; NR, normal range; SHBG, sex-hormone binding globulin; 17OHP, 17-hydroxyprogesterone.

^*a*^Transabdominal pelvic ultrasound.

Ethical approval was granted by the Western Sydney Local Health District Human Research Ethics Committee (HREC; AU RED HREC/16/WMEAD/53) and HREC, University of Sydney, Australia (HREC 10612; HREC 12502; HREC 13094).

### Statistics

*T* tests, ANOVA with Tukey's post-hoc analysis, Mann-Whitney's test, and Kruskal-Wallis with Dunn-Bonferroni post-hoc analysis were performed in SPSS (v26). *P* < .05 defined statistical significance.

### Label-free quantitative proteomic analysis

Mass spectrometry measured urine creatinine concentration (16, 17). For proteomic analysis, proteins were extracted using molecular weight centrifugal filtration cartridges (10 kDa), digested to peptides using trypsin (Promega, Southampton, UK) and desalted using C18-SPE (Agilent\) according to Hällqvist et al (16, 17). Peptides were analyzed using a 2D-NanoAquity liquid chromatography system (Waters). Samples were fractionated into ten fractions over 14 hours. Raw fractionated data were exported to Progenesis QI (Waters) and combined for analysis. Only proteins with ≥1 unique peptide per protein and an identification confidence score ≥15 were analyzed. To balance biological stringency against avoiding exclusion of potential biomarkers, 2 analyses were performed per study: a low stringency protein set (confidence score ≥15 and ≥1 unique peptide) and a high stringency set (confidence score ≥20 and ≥2 unique peptides).

Bioinformatic analysis was undertaken for proteins with a significant difference in abundance between PMOS and IR/control cohorts. Ingenuity Pathway Analysis (IPA; Qiagen) explored biological networks and identified enriched canonical pathways, biological processes, and upstream regulators, which are most closely associated with differentially expressed genes identified in discovery analysis, and predicted downstream effects on processes and disease. Gene Ontology annotation identified biological processes, molecular functions, and protein tissue expression using UniProtKB, GeneCards, a human gene database, and the Human Protein Atlas (18-22).

### Targeted proteomic analysis

Urine samples were analyzed with an in-house multiplexed targeted proteomic assay of 89 pro- and anti-inflammatory associated proteins, including cardiovascular and neuroinflammatory proteins, inflammatory cascade mediators e(g, heat shock proteins and cytokines), innate and adaptive immune mediators, and regulators of lipoprotein metabolism (16). The full methodology and protein list have been published at protocols.io (17, 23). For accurate quantitation, 15 μL of 30 ng/μL whole yeast enolase (Sigma) internal standard was added to 4 mL urine. Proteins were ultracentrifuged and digested to peptides using trypsin. After desalting, peptides were separated by reverse-phase chromatography over a 16-minute acetonitrile gradient. Each protein is represented by 1 or 2 unique peptide sequences and 2 to 4 quantifier and qualifier fragment ions. Peptides were analyzed on a Waters Acquity Ultra Performance Liquid Chromatography system coupled to a Xevo TQ-S mass spectrometer. Raw data were acquired using MassLynx v 4.1 (Waters) in multiple reaction monitoring mode and imported into Skyline v19 (MacCoss Lab Software) for processing. Protein–peptide sequences were obtained from www.uniprot.org, and settings for peptide detection and retention times were optimized using custom-synthesized peptides (Genscript) (18). Peak intensity data were normalized to a spiked yeast enolase.

### Data analysis

Normalized data were exported to Microsoft Excel (v16.58) and standardized to urinary creatinine. Data were analyzed in SIMCA (v15; Umetrics), SPSS, and GraphPad Prism (v9). ANOVA, and Student *t* test analyses were performed. The average fold-change in relative protein abundance was calculated to determine up- or downregulation of a protein, where >±1.5 was the minimum for the study, and a fold-change ±2 was more likely to indicate biological significance.

## Results

**Figure 1:**
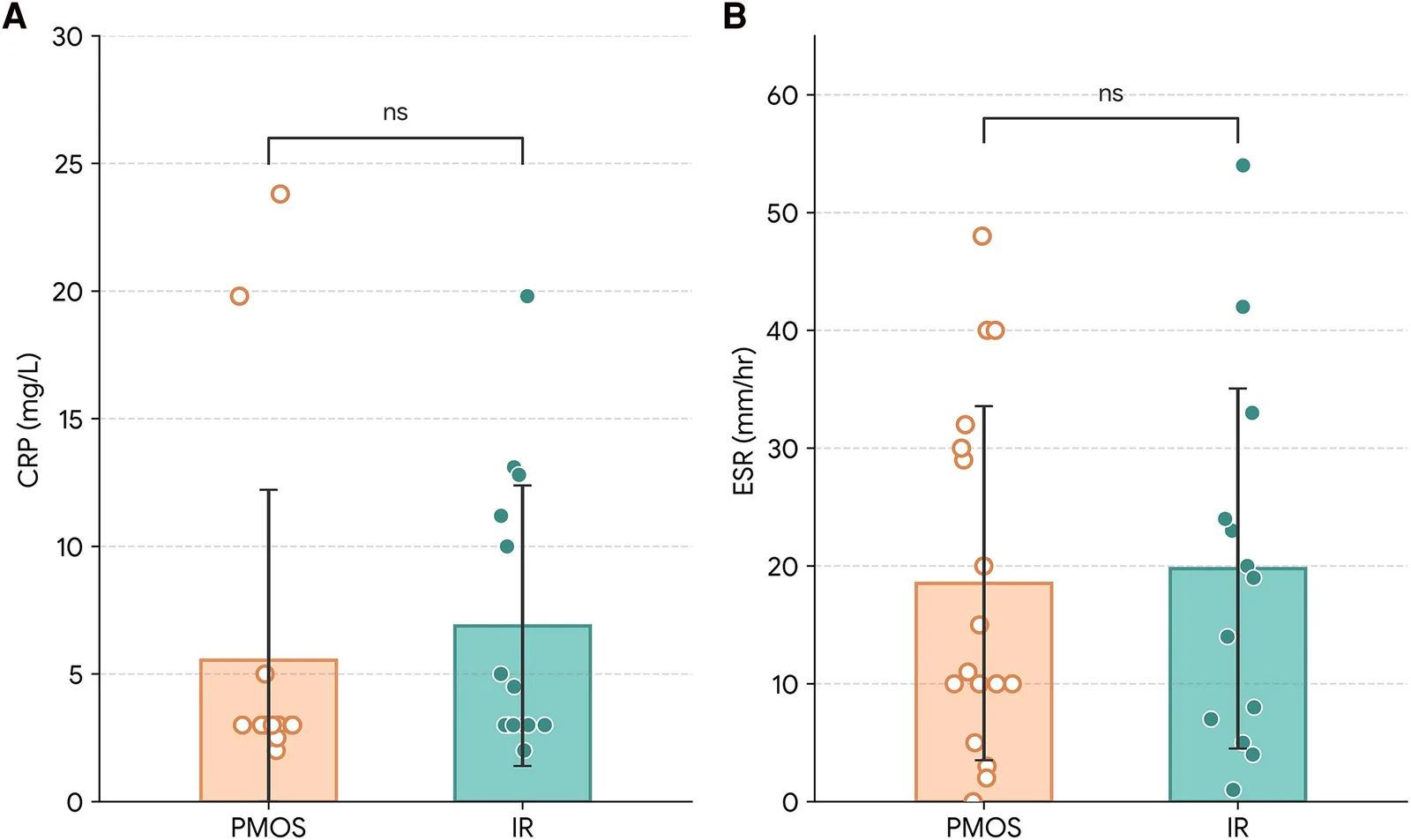
Bar scatter plots displaying univariate comparison of CRP and ESR in the adolescent PCOS (PMOS) and IR cohorts. Bars display the mean from each cohort. Scatter points denote individual values; error bars represent the SD. Abbreviations: CRP, C-reactive protein; ESR, erythrocyte sedimentation rate; IR, insulin resistance; ns, nonsignificant; PCOS, polycystic ovary syndrome; PMOS, polyendocrine metabolic ovarian syndrome.

Samples from adolescents with PMOS ± IR were compared with adolescents with IR only and healthy controls (16). Table 1 summarizes baseline clinical/biochemical data for all 40 participants, who were matched for age, menarcheal age, and postmenarcheal age. There was no significant difference in the HbA1c, Homeostatic Model Assessment for Insulin Resistance, body mass index, waist-to-height ratio, and body fat percentage between the PMOS and IR cohorts, meaning that PMOS participants also had IR and both cohorts had significant obesity. As such, differences in the proteome of these 2 cohorts are more likely to be due to PMOS rather than glucose or insulin metabolism and adiposity. Five participants reported occasional use of nonsteroidal anti-inflammatory drugs for dysmenorrhea, with no difference between cohorts. No participants were receiving insulin sensitizers, antidiabetic medications, antiandrogens, hormonal contraceptives, hormone replacement therapy, antihypertensives, or lipid-lowering medications at the time of sample collection. The traditional inflammatory markers, erythrocyte sedimentation rate (ESR) and C-reactive protein (CRP), demonstrated substantial overlap between cohorts and did not provide clear discrimination between PCOS, IR, and control participants (Table 1, Fig. 1).

### Biomarker discovery and pathway analyses: label-free quantitative proteomics to compare the urinary proteome in PMOS, insulin resistance, and control cohorts

Discovery proteomic analysis identified 3793 proteins, of which 314 were significantly and differentially expressed in the PMOS cohort in comparison to controls, and 397 in PMOS in comparison with IR. To identify the most clinically useful biomarkers, analysis focused on proteins that were differentially expressed in the PMOS cohort compared with both cohorts (n = 66), termed consensus proteins, forming a pool of candidate PMOS biomarkers. The majority of these had concordant directionality, but 3 were incongruent (eg, protein overexpressed in PMOS in comparison to IR) and underexpressed compared with controls (Figure SA, Table SA) (24).

### Identification of biological pathways and processes in PMOS

**Figure 2:**
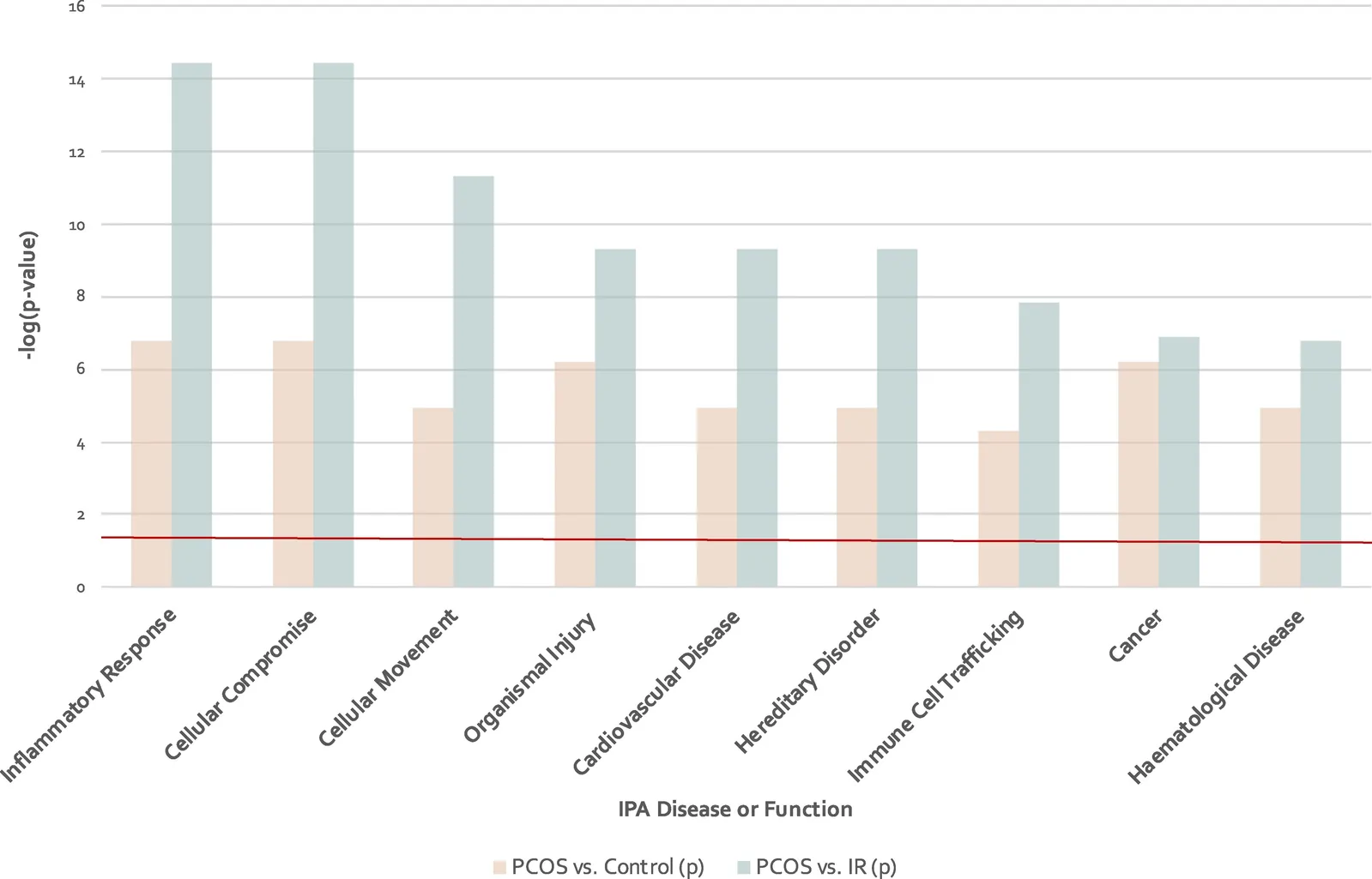
Consensus Ingenuity Pathway Analysis of diseases and functions. Each pair of bars represents a disease or function that was identified as significant in PCOS (PMOS) vs control and PCOS vs IR studies, listed in order of significance according to the PCOS vs IR study. The nonaxial horizontal line denotes significance (−log > 1.3, equivalent to *P* < .05). Abbreviations: IR, insulin resistance; PCOS, polycystic ovary syndrome; PMOS, polyendocrine metabolic ovarian syndrome.

IPA correlates genes/proteins and biological processes to indicate the clinical and biological relevance of identified biomarkers. Nine of the most significant processes differentially involved with PMOS compared with IR or control cohorts were common to both analyses (Fig. 2). Inflammatory response (*P* < .001) was the most significant. Other significant related processes included cellular compromise, organismal injury, cardiovascular diseases, immune cell trafficking, and hematological diseases. Stress response was the highest-ranked biological process associated with consensus proteins in gene ontology analysis, and immune system process and reproduction were within the top 10 biological processes.

**Figure 3:**
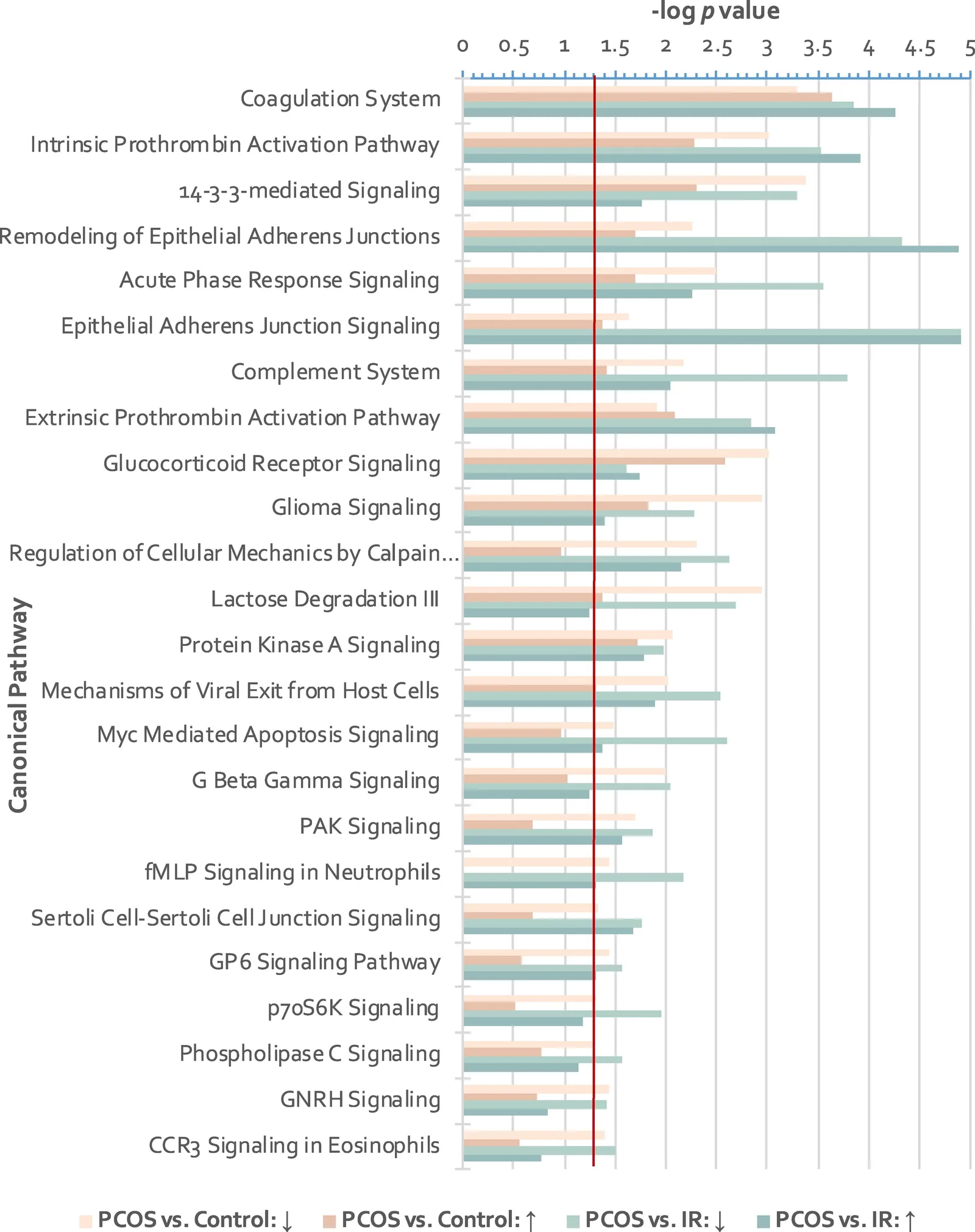
Consensus canonical pathways. Canonical pathway significance relates to how strongly identified proteins are associated with known biological pathways. Significance is expressed as −log *P* value on the *x* axis, where −log *P* > 1.3 = *P* < .05. Pathways displayed in order of the average ranking of the −log *P* value across all 4 stringency sets. Canonical pathways that were significant in at least 1 protein stringency set (⇡ = high stringency set [ie, confidence value >20, unique peptides >2]; ⇣ = low stringency set [ie, confidence value >15, unique peptides >1]) in PCOS (PMOS) vs control and PCOS vs IR studies. While proteins with higher confidence and unique peptide scores are more statistically robust, there are many lower-confidence and lower unique peptide proteins with high biological significance. Abbreviations: IR, insulin resistance; PCOS, polycystic ovary syndrome; PMOS, polyendocrine metabolic ovarian syndrome.

The IPA canonical pathway module illustrates the location of identified significant proteins within signaling pathways. Of the 645 differentially expressed proteins, 488 were imported into IPA, featuring in 1321 canonical pathways. There were 291 consensus pathways (ie, common to both PMOS vs control and PMOS vs IR analyses). Of these, 23 were significant (−log *P* value 1.31-4.90) (Fig. 3). The top consensus pathways were the coagulation system, intrinsic prothrombin activation pathway, 14-3-3–mediated signaling, remodeling of epithelial adherens junctions, and acute phase response signaling.

Individual proteins within canonical pathways were analyzed to identify potential biomarkers; 27 were in both PMOS/control and PMOS/IR analyses, and 21 were in significant pathways in both studies (Table SB) (24). Most proteins featured in more than 1 significant pathway (median, 2; range, 1-43), highlighting the interconnectedness of processes in PMOS.

Protein tissue expression was widespread, indicating that urinary proteins are reflective of systemic health and disease. Consensus proteins were highly expressed in adipose, liver, heart, and muscle. There was notable abundance in the female reproductive tract, including ovaries, and moderate expression in immune cells and some areas of the brain (Fig. SB) (24).

### Identifying clusters of biological functions in PMOS

Understanding biological clusters of proteins and pathways in IPA analysis may help identify mechanisms most relevant to PMOS. Almost half of all significant biological consensus pathways (10/23; 43%) and over one third (27/66; 41%) of consensus proteins were related to inflammatory (eg, acute phase signaling) and immune processes (eg, activated complement), including oxidative stress and the thrombotic and fibrinolytic systems. (Fig. SC) (24). Many proteins were mediators of complement, coagulation, apoptosis cascades, or proinflammatory cytokines.

The second-most abundant biological cluster concerned cytoskeleton reorganization, remodeling, apoptosis, and folliculogenesis, involving one third (8/23; 35%) of pathways and one third (24/66; 37%) of proteins. Glucose homeostasis/IR, hyperandrogenism, and neuroendocrine and metabolic pathways were associated with one third (n = 21/66; 32%) of proteins and almost a quarter of pathways (5/23; 22%). One fifth (12/66; 18%) of proteins and one pathway were related to neurological, neuroplasticity, neurodegenerative, and neuropsychiatric processes.

These novel exploratory findings provide detailed insight into this complex multisystem disease and implicate inflammatory proteins and pathways at the heart of PMOS pathophysiology in adolescents. We have demonstrated that the strongest discriminators between PMOS, IR, and healthy controls were differences in inflammatory pathways and inflammatory proteins. Despite overlapping inflammatory and metabolic characteristics, PMOS and IR exhibit distinct inflammatory proteomic profiles, supporting the presence of condition-specific inflammation. Consequently, targeted proteomic analysis was undertaken to identify the specific proteins driving these differences and to determine which inflammatory proteins were up- or downregulated in PMOS.

### Validating urinary proteomic biomarkers and inflammatory processes

**Figure 4:**
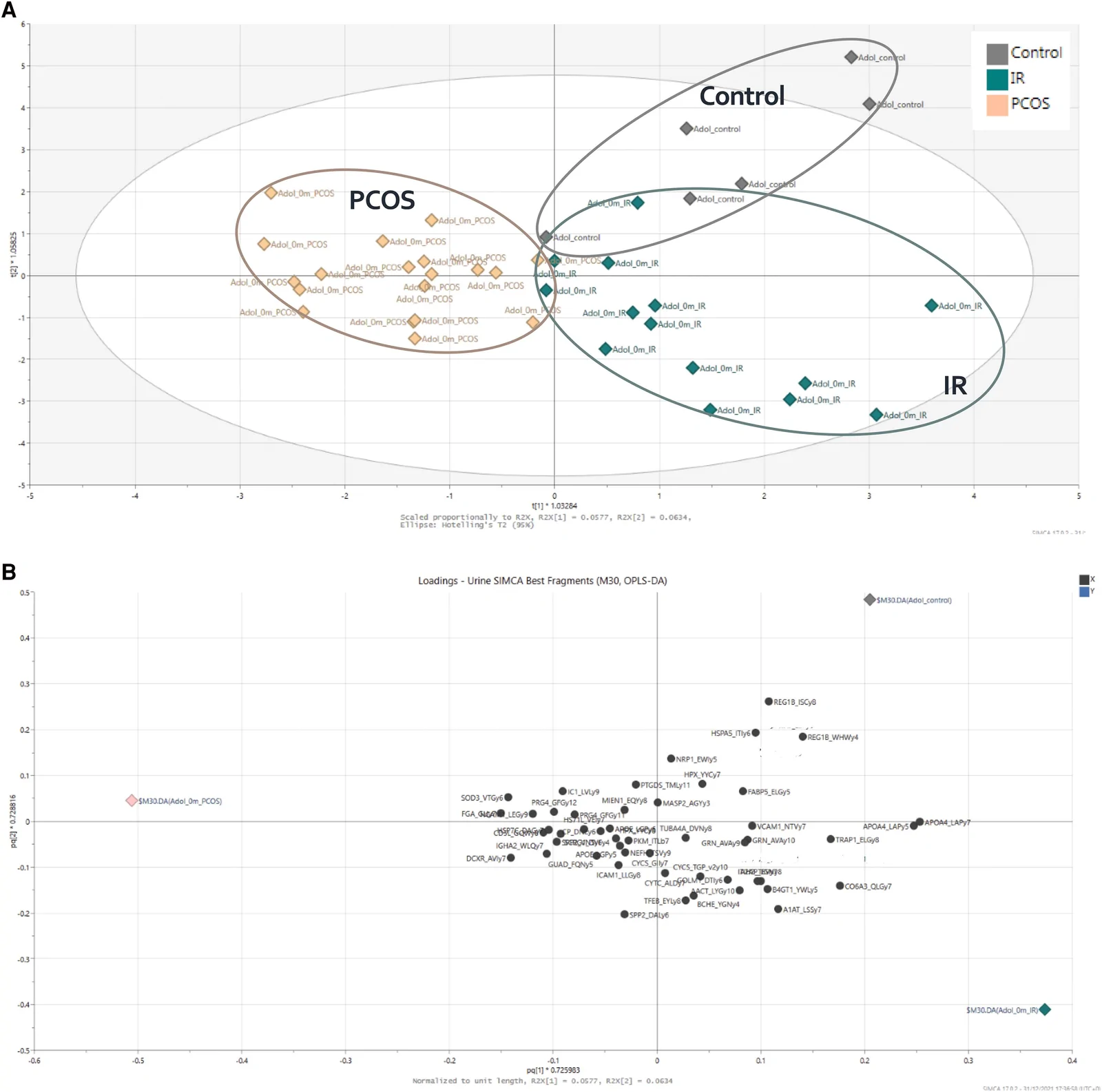
SIMCA OPLS-DA plots for all measurable inflammatory proteins in all cohorts. (A) OPLS-DA score scatterplot. Data points represent individual participants in the following cohorts: PCOS, IR, and control. The ovals highlight the separation between cohorts. There is good separation between the PCOS (PMOS) cohort compared with other cohorts, indicating quantifiable differences in these inflammatory proteomes. Some overlap remains between the control and IR cohorts, indicating more subtle differences in the inflammatory profile of these 2 cohorts. (B) OPLS-DA loading scatter plot. X variables (circles) represent individual proteins (labeled with gene names, peptide sequence, and ion fragment). Y variables (diamonds) represent the cohorts as a collective data point: PCOS (PMOS) (far left), IR (bottom right), and control (top right) cohorts. Proteins in closest proximity to Y or cohort variables are those that are the most significantly expressed and have the greatest abundance in that cohort and therefore are most likely to be driving the differences in the inflammatory proteome. Abbreviations: IR, insulin resistance; OPLS-DA, orthogonal partial least squares discriminant analysis; PCOS, polycystic ovary syndrome; PMOS, polyendocrine metabolic ovarian syndrome.

Findings were validated with a wide range (n = 89) of inflammatory-associated biomarkers in urine, using a targeted proteomic mass spectrometry assay (23). Given the abundance of proteins associated with neurological and neuropsychiatric conditions in this study, alongside mounting evidence linking PMOS and mood disorders, the panel included neuroplasticity, neurodegeneration, and neuroinflammation biomarkers (25-27). All 89 proteins were detected in urine. Proteins of low abundance were excluded due to unreliability (n = 47). Multivariate analysis of the 42 remaining proteins was undertaken in SIMCA. Principal component analysis score scatter plots demonstrated good sample integrity but showed no discernible separation between the 3 cohorts, indicating that significant differences were not identified when considering the inflammatory proteome collectively, which is common in omic analysis. However, orthogonal partial least squares discriminant analysis (OPLS-DA) identified clustering within, and separation between cohorts, particularly in PMOS compared with both other cohorts (Fig. 4A). These subtle but significant and quantifiable differences between inflammatory proteomes were likely to be driven by a smaller number of proteins. Therefore, individual proteins were interrogated using univariate analyses.

The OPLS-DA loading scatter plot (Fig. 4B) displays individual proteins that are most likely to be driving differences in inflammatory profiles and are candidate biomarkers. Fibrinogen α chain, superoxide dismutase-3 (SOD3), dicarbonyl/L-xylulose reductase (DCXR), neural cell adhesion molecule-1 (NCAM1), CD5 molecule like (CD5L), and immunoglobulin heavy constant α 2 were the most significantly associated with the PMOS cohort. Several proteins were downregulated in the PMOS cohort and upregulated in the IR/control cohort, including apolipoprotein A4 (APOA4), collagen type VI alpha-3 chain (COL6A3), TNF-receptor associated protein 1 (TRAP1), and regenerating family member-1 beta (REG1B).

### Comparing the inflammatory proteome in PMOS and controls

**Figure 5:**
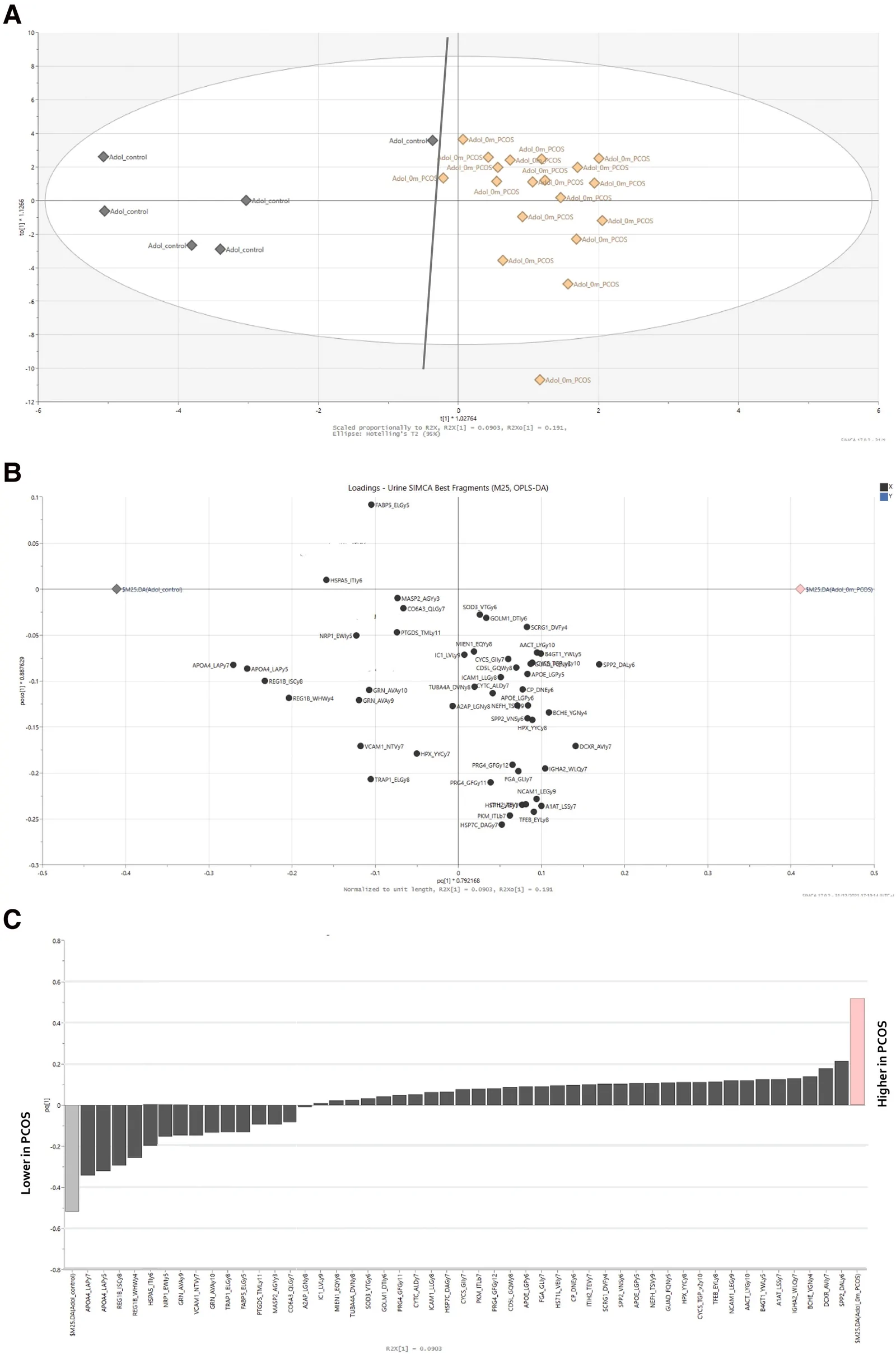
SIMCA plots for measurable inflammatory proteins in PCOS (PMOS) and control cohorts. Individual participants are denoted by data points: control (left, n = 6) and PCOS (PMOS) (right, n = 19). The vertical line in panel A highlights the clear separation between cohorts. When you overlay panel B onto panel A, individual proteins most responsible for variations in the observations can be identified. Proteins closest to the far right diamond data point (PCOS) and far left diamond data point (controls) are most responsible for driving the difference between the cohorts. Proteins in panel B are denoted by circle data points (labeled according to gene name, peptide sequence, fragment ion). (C) OPLS-DA loading column plot demonstrating the proteins driving the difference between control and PCOS (PMOS) cohorts. Proteins are labeled according to gene name, peptide sequence, and fragment ion on the *x* axis. Cohorts are labeled and represented by bars at the far right (PCOS/PMOS) and far left (control). Proteins are represented by bars between the cohort bars. Bar height shows loadings and which proteins are most up- or downregulated in PCOS and control cohorts. Proteins that are further to the right have an increased abundance and significance in the PCOS cohort. Proteins on the left have a decreased abundance and significance in the PCOS cohort from SIMCA. Abbreviations: OPLS-DA, orthogonal partial least squares discriminant analysis; PCOS, polycystic ovary syndrome; PMOS, polyendocrine metabolic ovarian syndrome.

SIMCA OPLS-DA (Fig. 5) demonstrated good separation in the inflammatory profiles between these 2 cohorts, and therefore likely differences in disease mechanisms. The loading column plot (Fig. 5C) quantitatively displays the relative abundance of each protein. Secreted phosphoprotein-2 (SPP2), DCXR, butyrylcholinesterase, IGHA2, and alpha-1-antitrypsin (A1AT) were most associated with PMOS. Proteins that were most significantly downregulated in the PMOS cohort were APOA4, REG1B, heat shock protein family A member 5 (HSPA5), neuropilin-1 (NRP1), and granulin precursor.

**Figure 6:**
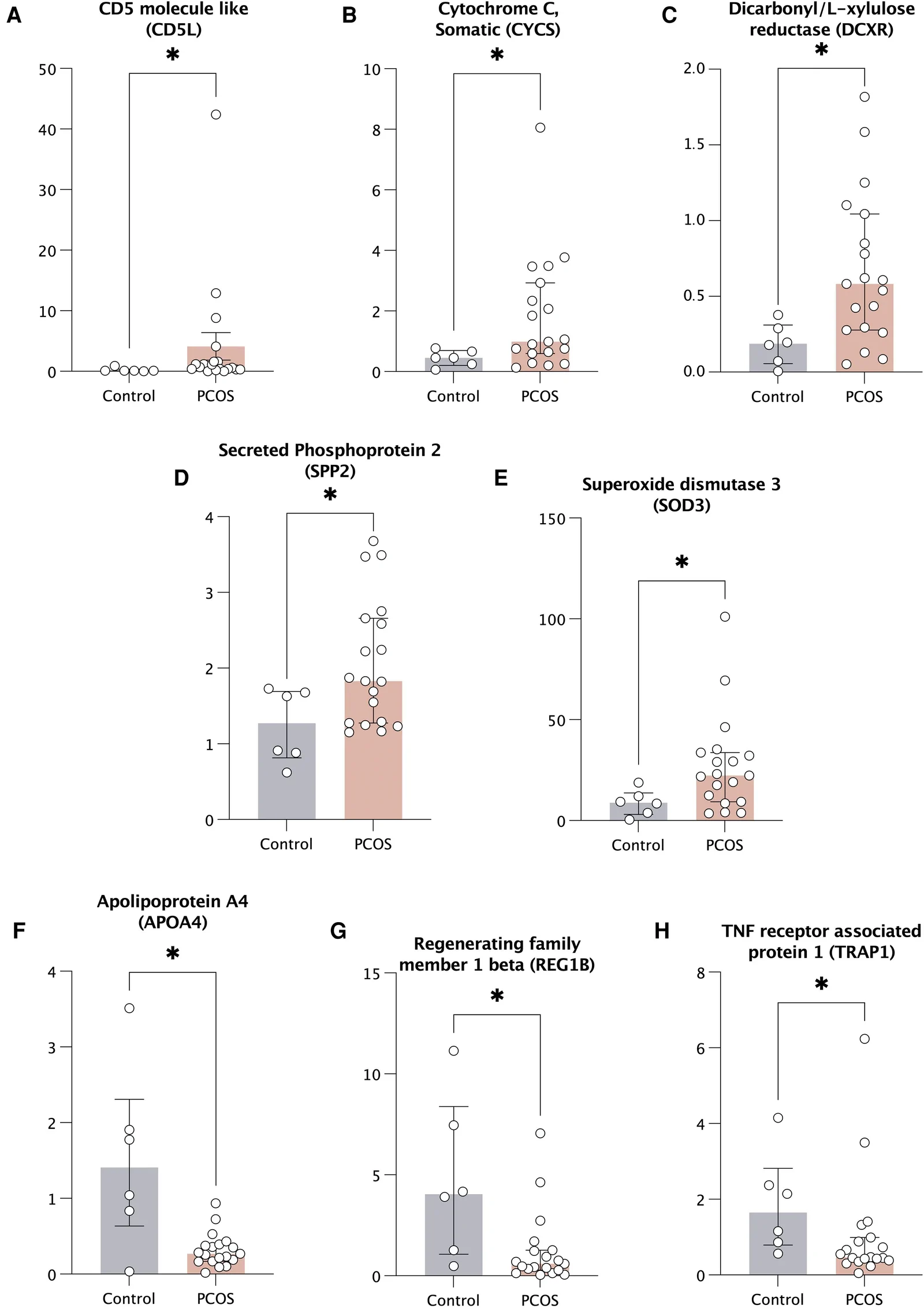
Bar scatterplots displaying univariate comparison of proteins that are significantly different in the adolescent PCOS (PMOS) and healthy control cohorts. Bars display the median of the normalized abundance from each cohort. Scatter points denote individual values; error bars represent the interquartile range (IQR). Panels A to E display the 5 significant (*P* < .05) proteins that were upregulated in the PCOS (PMOS) cohort. Panels F to H display the 3 significant proteins that were downregulated in the PCOS cohort. *P < .05. Abbreviations: PCOS, polycystic ovary syndrome; PMOS, polyendocrine metabolic ovarian syndrome.

Univariate analyses comparing PMOS and control cohorts, identified 8 significantly different proteins (Fig. 6). Figure 6A-6E displays the 5 upregulated proteins in PMOS; CD5L (*P* = .014), cytochrome C, somatic (*P* = .036), DCXR (*P* = .013), SPP2 (*P* = .026), and SOD3 (*P* = .026). Figure 6F-6H displays the 3 downregulated proteins: APOA4 (*P* = .016), REG1B (*P* = .016), and TRAP1 (*P* = .031).

### Comparing the inflammatory proteome in PMOS and insulin resistance

**Figure 7:**
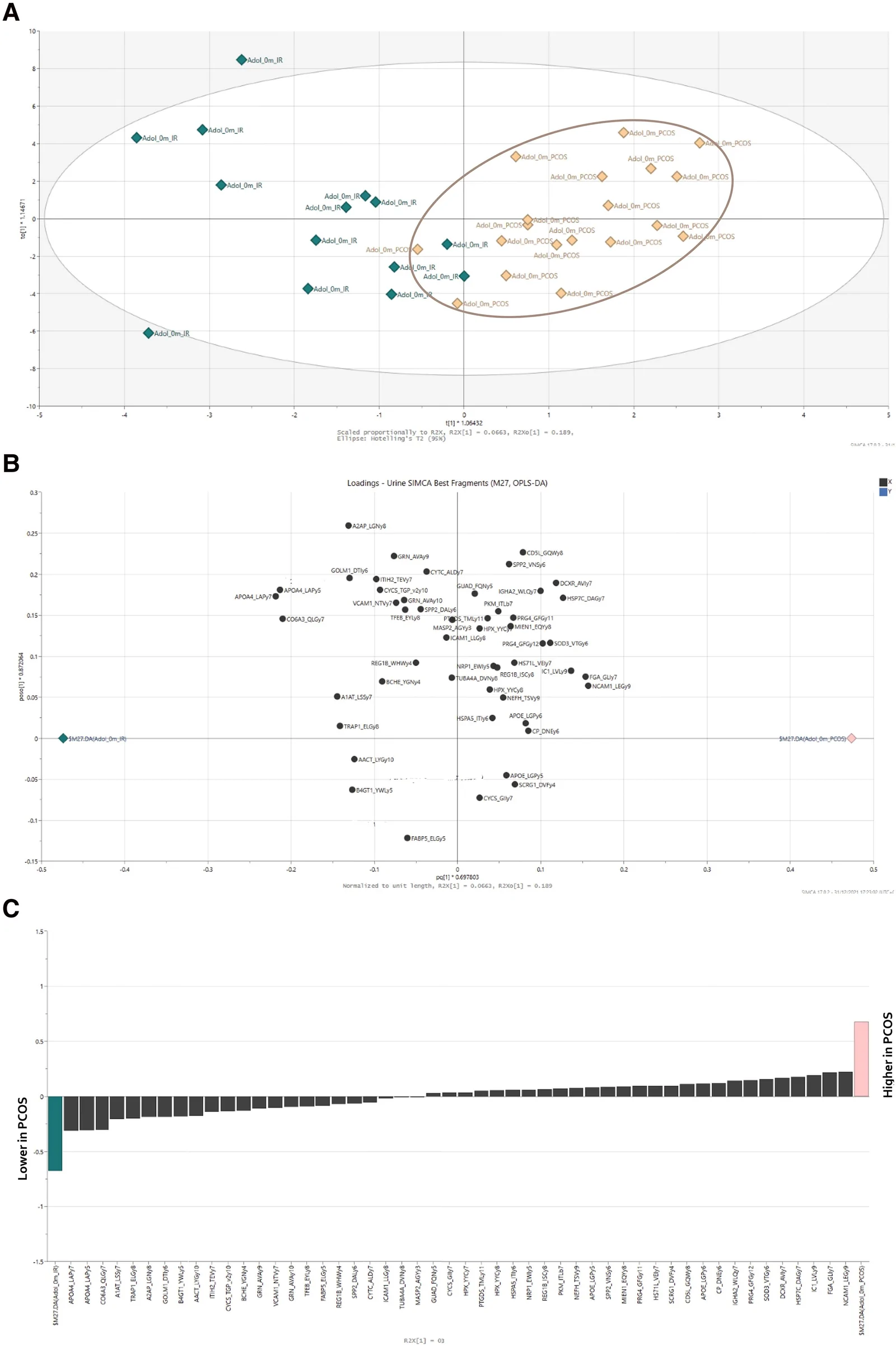
SIMCA plots for measurable inflammatory proteins in adolescent PCOS and IR cohorts. (A) Individual participants denoted by colored data points: PCOS (right, n = 19) and IR (left, n = 15). The oval outline highlights the partial separation of the PCOS cohort. (B) Proteins closest to the far right diamond data point (PCOS) and far left diamond data point (IR) are most responsible for driving the difference between the cohorts. Proteins labeled according to gene name, peptide sequence, and fragment ion. (C) OPLS-DA loading column plot demonstrating the proteins driving the difference between cohorts. Proteins are labeled according to gene name, peptide sequence, and fragment ion on the *x* axis. Bar height represents loadings. Cohorts are labeled and represented by bars at the far right (PCOS) and far left (IR). Proteins are represented by bars between cohort bars. Proteins that are further to the right have an increased abundance and significance in the PCOS cohort, whereas proteins on the left have a decreased abundance and significance in the PCOS cohort from SIMCA multivariate analysis. Abbreviations: IR, insulin resistance; OPLS-DA, orthogonal partial least squares discriminant analysis; PCOS, polycystic ovary syndrome; PMOS, polyendocrine metabolic ovarian syndrome.

SIMCA multivariate analysis demonstrated almost complete separation between cohorts in OPLS-DA, again indicating differences in disease mechanisms (Fig. 7). NCAM1, fibrinogen α chain, H19/IGF2 imprinting control region, heat shock protein family A member-8 (HSP7C/HSPA8), DCXR, and SOD3 were the most abundant and significantly associated with the PMOS cohort. APOA4, COL6A3, A1AT, and TRAP1 were significantly downregulated.

**Figure 8:**
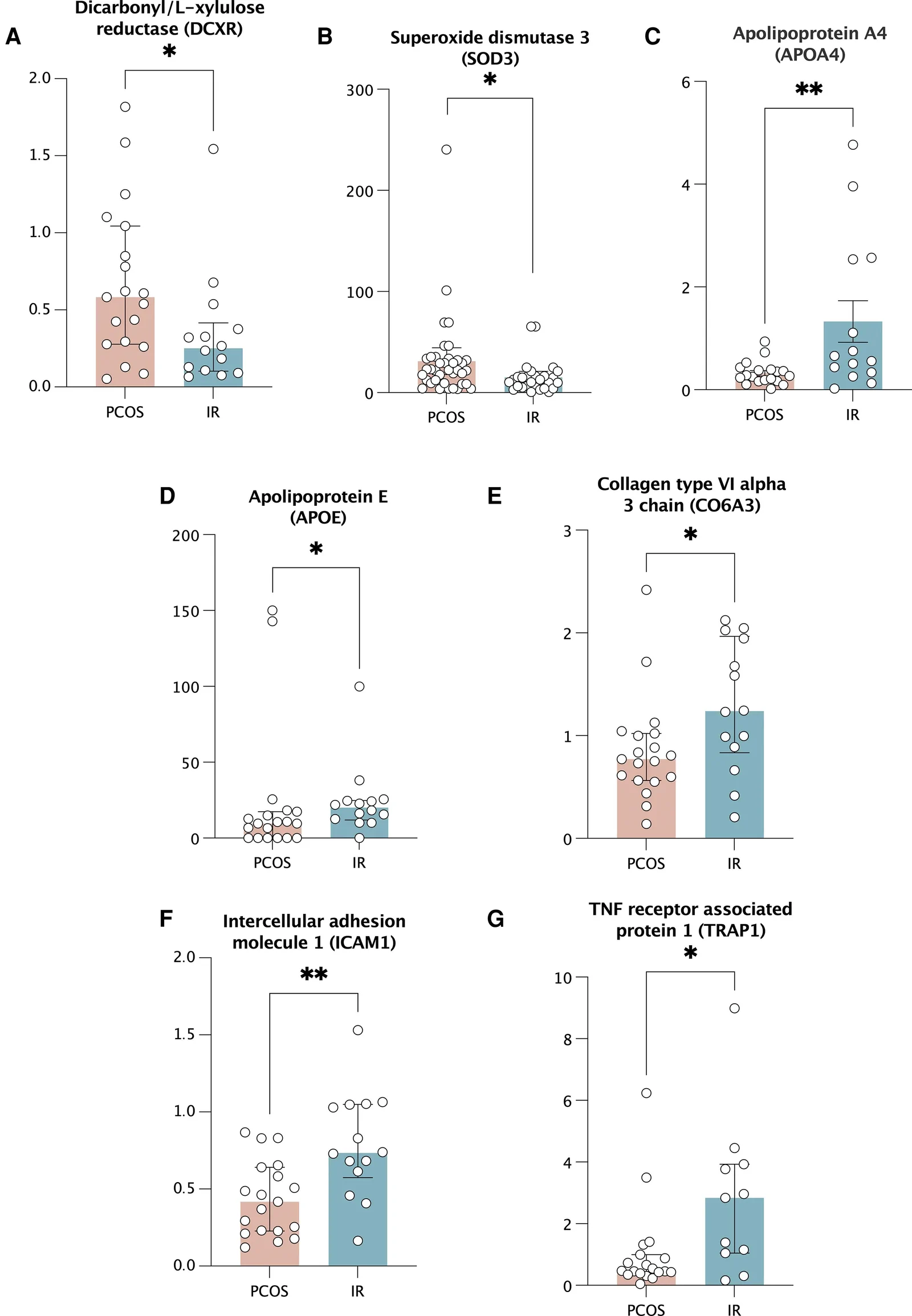
Bar scatter plots displaying univariate comparison of proteins that are significantly different between the adolescent PCOS (PMOS) and IR cohorts. Bars display the median of the normalized abundance from each cohort. Scatter points denote individual values; error bars represent the IQR. Panels A and B display the 2 significant (*P* < .05) proteins that were upregulated in the PCOS cohort. Panels C to G display the 5 significant proteins that were downregulated in the PCOS cohort. *P < .05, **P < .01. Abbreviations: IQR, interquartile range; PCOS, polycystic ovary syndrome; PMOS, polyendocrine metabolic ovarian syndrome.

Univariate comparison between PMOS and IR cohorts identified 7 significantly different proteins (Fig. 8). DCXR (*P* = .029) and SOD3 (*P* = .049) were upregulated in the PMOS cohort (Figs. 8A and 8B). APOA4 (*P* = .011), apolipoprotein E (APOE; *P* = .030), COL6A3 (*P* = .041), intercellular adhesion molecule-1 (ICAM1; *P* = .004), and TRAP1 (*P* = .015), were downregulated (Fig. 8C-8G).

### Identification of novel inflammatory biomarkers in PMOS

**Figure 9:**
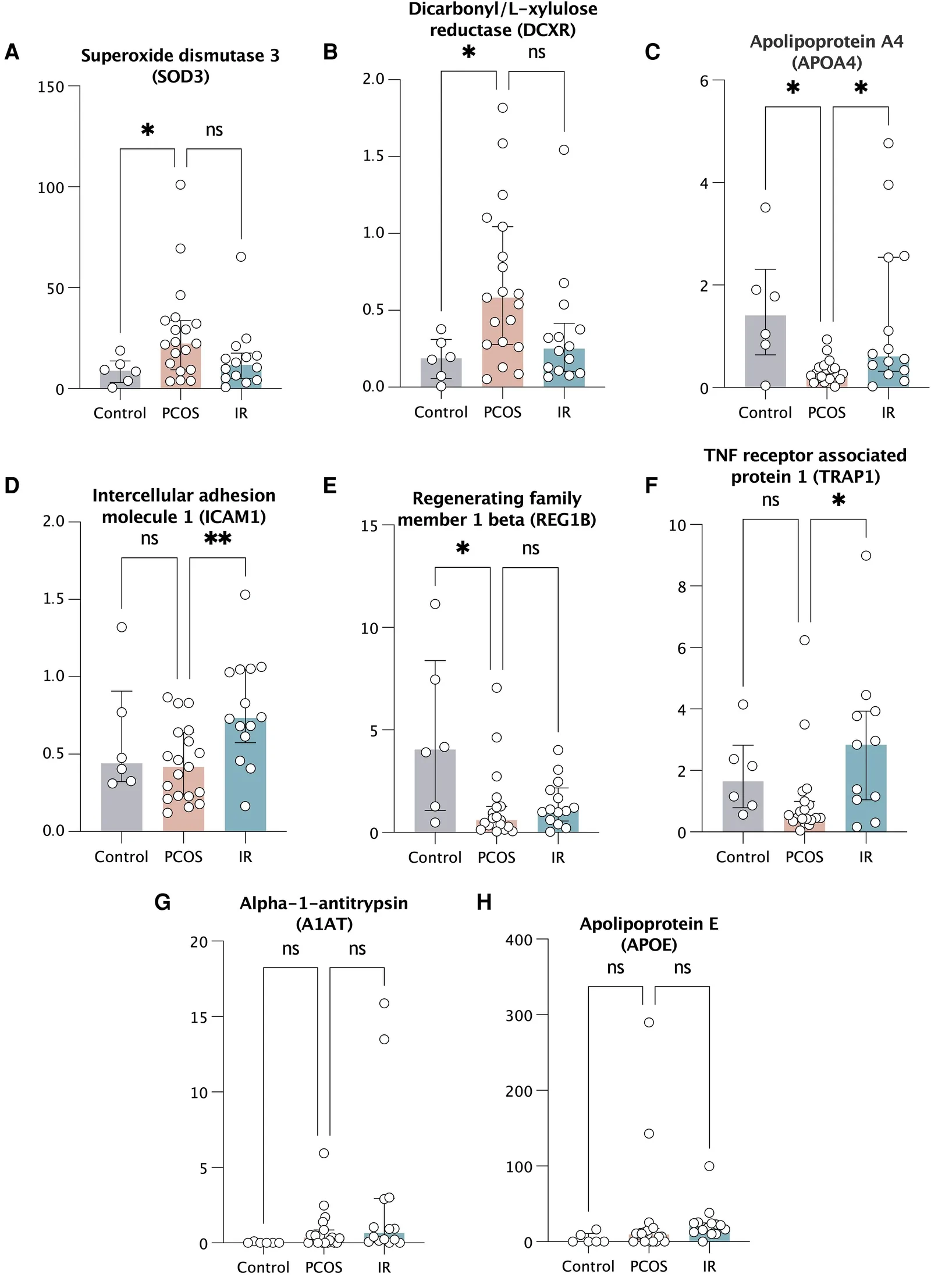
Bar scatterplots displaying multivariate comparison of proteins that are significantly different in the adolescent PCOS (PMOS), IR, and healthy control cohorts. Bars display the median of the normalized abundance from each cohort. Scatter points denote individual values; error bars represent the IQR. Panels A and B display the 2 significant (*P* < .05) proteins that were upregulated in the PCOS (PMOS) cohort in comparison to both other cohorts. Panels C to F display the 4 significant proteins that were downregulated in the PCOS cohort. Panels G and H display the 2 proteins that were upregulated in PCOS in comparison 1 cohort and downregulated in comparison to the other. *P < .05, **P < .01, ns = nonsignificant in post-hoc analysis of the PCOS cohort in comparison to IR or control cohorts. Abbreviations: IQR, interquartile range; IR, insulin resistance; PCOS, polycystic ovary syndrome; PMOS, polyendocrine metabolic ovarian syndrome.

Multivariate analysis of PMOS, IR, and control cohorts identified 8 significantly different proteins in 1 cohort in comparison to others (Fig. 9). All have been addressed in the previous discussion. Two proteins were upregulated in PMOS (Fig. 9A and 9B): SOD3 (*P* = .030) and DCXR (*P* = .014). Four proteins were downregulated (Fig. 9B-9F): APOA4 (*P* = .008), ICAM1 (*P* = .017), REG1B (*P* = .027), and TRAP1 (*P* = .018). A further 2 proteins were upregulated in PMOS in comparison to 1 cohort and downregulated compared with the other (Fig. 9G-9H): APOE (*P* = .007) and A1AT (*P* = .022), limiting utility as biomarkers.

## Discussion

We have mapped the proteome in adolescent PMOS using discovery proteomic analysis, providing insight into its molecular underpinnings and confirming urine as a valuable biological matrix. Of 3793 identified proteins, 66 were differentially expressed in PMOS compared with the control and IR cohorts. These proteins and biological pathways were related to cytoskeletal remodeling and folliculogenesis, apoptosis, insulin, metabolic and neuroendocrine signaling, and neurological and neuropsychiatric disorders. Many of these are established or emerging areas in PMOS, but these data provide granularity to the existing literature, particularly during adolescence (16, 25-28). The major finding of this discovery analysis was the significant abundance of immune and inflammatory proteins, suggesting that these may be central to the pathophysiology of PMOS. Individual proteins and pathways from this exploratory study are discussed by Gunn et al (16).

Emerging literature suggests PMOS is a chronic low-grade inflammatory state. However, disentangling PMOS-specific inflammatory patterns (vs obesity and IR) is challenging (29-31). Previous studies using targeted assays and invasive matrices, including blood and follicular fluid, have demonstrated elevated inflammatory markers in PMOS but are limited by the restricted number of biomarkers tested, none evaluated noninvasive matrices, and only 3 focused on adolescents (29-32). In this study, traditional inflammatory markers, including CRP and ESR, did not significantly differ between cohorts, highlighting the value of novel inflammatory biomarkers to distinguish PMOS from IR and healthy controls.

Our targeted inflammatory proteomic analysis internally validated the association between PMOS and inflammation, demonstrating broad alterations across multiple inflammatory-associated proteins. Given the inflammatory component in IR, it is unsurprising that differences between the PMOS and healthy control inflammatory proteomes were greater than those observed between PMOS and IR. However, distinct inflammatory proteomic profiles were identified between PMOS and both comparison groups. Additionally, differential expression of specific proteins supports the presence of a PMOS-specific inflammatory signature and highlights potential targets for future biomarker development and therapeutic intervention. The clustering of participants within cohorts indicated consistent protein expression patterns, suggesting that these findings may be representative of similar clinical populations, although confirmation in larger and more diverse cohorts is required.

The cross-sectional nature of the study precludes conclusions regarding causality, and it is possible that the observed inflammatory signature represents a consequence of underlying genetic, developmental, endocrine, or metabolic disturbances that contribute to PCOS, rather than a primary driver of disease onset. Regardless of whether inflammatory changes are causal or secondary, their prominence in adolescence suggests they may contribute to disease expression, progression, or long-term comorbidity risk, representing clinically relevant biomarkers or therapeutic targets.

### Exploring inflammatory processes and PMOS: targeted proteomic analysis

Across SIMCA, univariate, and multivariate targeted inflammatory analyses comparing adolescent PMOS, control, and IR cohorts, 11 proteins were significantly elevated in PMOS, and 9 proteins were downregulated. These are potential biomarkers. Six proteins with concordant differences across both comparator groups are of most value (ie, up- or downregulated in PMOS compared with both IR and control cohorts); SOD3 and DCXR were upregulated in PMOS, and APOA4, ICAM1, REG1B and TRAP1 were downregulated concordant proteins.

### Oxidative stress, apoptosis, and neuroinflammation

Oxidative stress biomarkers were heavily implicated. These can impact folliculogenesis and promote anovulation and drive abnormal oocyte meiotic spindle formation (33). Oxidative stress promotes conditions associated with PMOS including diabetes, abdominal adiposity, and cardiometabolic dysfunction. Apoptosis and autophagy are enhanced in granulosa cells in PMOS (34). Cytochrome C, somatic, upregulated in the PMOS cohort, has a role in triggering apoptosis and oxidative stress and has been identified in the granulosa cells of women with obesity (35). Immunoglobulin heavy constant α 2 promotes oxidative stress and the inflammatory cascade and was significantly associated with PMOS in this study (18, 21). Interestingly, antioxidant SOD3, a concordant protein, was elevated in the PMOS cohort. Studies have demonstrated that increased antioxidant activity in follicular fluid is associated with decreased oocyte fertilization in PMOS, and its abundance may reflect an attempt to protect oocytes from reactive oxygen species and apoptosis (36). Oxidative stress and antioxidants appear to disrupt and improve ovarian function, respectively; determining the effect of antioxidant therapy in PMOS is key (33).

Two downregulated proteins are related to vascular endothelial growth factor (VEGF) pathways—NRP1 and concordant protein TRAP1. NRP1 regulates vascular endothelial growth factor–induced ovarian angiogenesis and may have a role in inducing apoptosis (18, 21). Dysregulation of ovarian angiogenesis can impair follicular cell function, decrease ovulation, and increase small antral follicle formation. NRP1 downregulation is linked to PMOS (37). TRAP1 is a heat shock protein (HSP), protecting cells from oxidative stress and apoptosis (38). Two other stress response HSPs were identified; HSP7C/HSPA8 and HSPA5. HSP70 proteins have been correlated to testosterone and inflammatory markers in rat PMOS studies, with greater ovarian tissue expression and decreased serum expression (39). HSP70 proteins as drug targets for systemic and neuroinflammatory disorders, has been explored and may be a novel area of development in PMOS (40).

There is notable expression of other neuroinflammatory and neurodegenerative proteins. granulin precursor, downregulated in this study, has roles in anti-inflammation and angiogenesis regulation. Loss of expression is associated with neurodegeneration and neuroinflammation (41). NCAM1, upregulated in the PMOS cohort, has roles in cell-matrix interactions, neurogenesis regulation, and immune cell expansion and was upregulated in granulosa cells in PMOS (42). NCAM1's expression is modulated by transforming growth factor-beta proteins, of which anti-Müllerian hormone is one, and is involved in triggering MAPK and PI3 K cascades, which have roles in apoptosis and insulin signaling (18, 21, 43).

REG1B, another concordant protein, was downregulated in PMOS and has purported roles in pancreatic islet cell regeneration and neuronal sprouting in the brain (21). The link between PMOS, neuropsychology and neurodegeneration is rapidly developing, and thought to be mediated through inflammatory pathways and IR (25-28).

### Glucose homeostasis and dyslipidemia

Glucose homeostasis and insulin signaling are intrinsically linked with PMOS. Two related proteins were upregulated in PMOS, concordant protein DCXR and butyrylcholinesterase, which is implicated in ghrelin regulation, promoting food intake, obesity, and IR (21). Butyrylcholinesterase also has roles in lipid metabolism and neurodegeneration and small molecule detoxification.

Dyslipidemia is a central PMOS comorbidity. Two other upregulated proteins are involved in lipid metabolism: SPP2 and CD5L. The latter stimulates lipolysis and is associated with atherosclerosis and obesity-associated adipose inflammation (18, 21). ICAM1, a concordant protein and proinflammatory/proatherogenic cytokine associated with PMOS and IR, was downregulated in PMOS compared with IR, reflecting increased expression in the IR state (18, 21). Two key apolipoproteins were identified: APOE, essential for lipoprotein breakdown and associated with PMOS, metabolic dysfunction, neurodegeneration, and able to interact with androgen receptors (44), and APOA4, a concordant protein and lipid-binding protein and HDL component, which was significantly downregulated in PMOS. It has wide-ranging roles related to platelet aggregation and thrombosis, antiatherosclerosis, glucose homeostasis, and hypothalamic inhibition of food intake. Hemostatic and fibrinolytic abnormalities are increasingly linked to PMOS pathophysiology, reinforced by our study findings (45). APOA4 was increased in follicular fluid in PMOS, which may have a role in disrupting ovulation (46). However, other studies have shown that APOA4 is protective against diabetes and atherosclerosis and, when downregulated, can promote systemic metabolic dysfunction (47). There may be differential expression in the ovary and urine, and suppression of APOA4 in urine and perhaps serum may be a marker of PMOS. Confirming identified biomarkers in blood, in addition to urine, may further enhance their clinical utility and should be considered in future validation studies.

### Strengths and limitations

Limitations of this study include the relatively small sample size, potentially limiting the applicability of findings to wider populations. Given the large number of investigations requested for participants, some parameters were not obtained for all. This is particularly true for the control cohort, where pelvic ultrasound and OGTT's were not possible due to the practical constraints of community-based recruitment and the need to minimize invasive testing in healthy adolescents. However, controls underwent health screening, had normal body mass index and metabolic anthropometric parameters, and had no clinical or biochemical features suggestive of PMOS. Most adolescents referred to tertiary endocrine and gynecology services had overweight/obesity, meaning that few nonobese participants with PMOS or IR were recruited, reflecting the metabolic characteristics of adolescents referred to tertiary specialist services. Future studies should seek to include a subset of non-overweight/obese adolescents with PMOS to better understand the proteomic profile in those with a lean PMOS phenotype, in addition to an overweight/obese non-PMOS control cohort, to disentangle the role of obesity. Validation of discovery findings was performed with a larger cohort recruited from the same clinical setting. Future studies should provide external validation in a larger, geographically distinct cohort. Although this panel measured a wide range of inflammatory-associated proteins, findings are restricted to the proteins included in the targeted panel. Strengths include this study's unique focus on adolescents, with comparison made to both control and IR cohorts. Other strengths include confirming biomarkers in urine, high proteomic instrument sensitivity, and powerful multivariate and bioinformatic analyses.

## Conclusion

Discovery urine proteomics mapped the adolescent proteome, identifying inflammatory/immune processes as the most abundant in PMOS in our exploratory, hypothesis-generating study. Bioinformatic analysis highlighted the interrelation and complexity of pathways underpinning PMOS. Building on our discovery proteomic findings, which identified inflammatory pathways as key discriminators between PMOS and insulin resistance, targeted proteomic analysis revealed distinct patterns of inflammatory protein dysregulation, supporting unique inflammatory signatures in PMOS. Significant differences were identified in 20 proteins in PMOS vs IR and controls. Six of these are more robust candidate biomarkers for PMOS in adolescence. Noteworthy functions include oxidative stress, neuroinflammation and neurodegeneration. Regardless of its causal role, the early prominence of inflammation in PMOS highlights inflammatory pathways as potential biomarkers and therapeutic targets, with adolescence representing a key window for early intervention to improve the pro-inflammatory state. Future studies should validate these findings in larger external cohorts and employ longitudinal designs to determine the temporal relationship between inflammatory proteomic changes and PMOS across the reproductive lifespan.

## Data Availability

Additional data are provided in the Supplementary Information (24). Other data presented in this study are available from the corresponding author upon reasonable request.
